# (*E*,*E*)-2,5-Bis(4-*tert*-butyl­benzyl­idene)­cyclo­penta­none

**DOI:** 10.1107/S1600536808025543

**Published:** 2008-08-13

**Authors:** Jian Wei, Guang Liang, Yuhong Gai, Jingmei Lu

**Affiliations:** a College of Life Science, Northeast Normal University, Changchun, Jilin Province 130024, People’s Republic of China; b College of Life Science, Changchun Normal University, Changchun, Jilin Province 130017, People’s Republic of China; c College of Agriculture, Jilin Agricultural University, Changchun, Jilin Province 130118, People’s Republic of China

## Abstract

The asymmetric unit of the title compound, C_27_H_32_O, contains two and a half mol­ecules. In the crystal structure, one of the mol­ecules lies on a crystallographic twofold rotation axis. The dihedral angles between the benzene rings are 12.17 (6), 16.29 (11) and 51.24 (8)° for the three molecules. The dihedral angles between the benzene rings of each molecule in the asymmetric unit are 12.17 (6) and 16.29 (11)°

## Related literature

For related literature, see: Began *et al.* (1999 ▶); Kawamori *et al.* (1999 ▶); Liang *et al.* (2008 ▶); Liang, Tian *et al.* (2007 ▶); Liang, Yang *et al.* (2007 ▶); Livingstone & Walker (2003 ▶); Poorichaya *et al.* (2007 ▶); Saiah (2008 ▶).
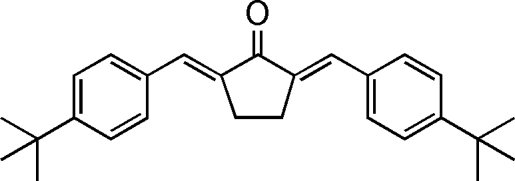


## Experimental

### 

#### Crystal data

C_27_H_32_O
*M* *_r_* = 372.53
Monoclinic, 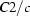

*a* = 41.246 (7) Å
*b* = 6.2930 (10) Å
*c* = 43.001 (7) Å
β = 94.243 (4)°
*V* = 11131 (3) Å^3^
*Z* = 20
Mo *K*α radiation
μ = 0.07 mm^−1^
*T* = 293 (2) K
0.50 × 0.41 × 0.18 mm

#### Data collection

Bruker SMART CCD diffractometer
Absorption correction: multi-scan (*SADABS*; Bruker, 2002 ▶) *T*_min_ = 0.761, *T*_max_ = 1.000 (expected range = 0.752–0.989)
28046 measured reflections
10373 independent reflections
4318 reflections with *I* > 2σ(*I*)
*R*_int_ = 0.128

#### Refinement

*R*[*F* ^2^ > 2σ(*F* ^2^)] = 0.068
*wR*(*F* ^2^) = 0.180
*S* = 0.86
10373 reflections
648 parameters
H-atom parameters constrained
Δρ_max_ = 0.20 e Å^−3^
Δρ_min_ = −0.18 e Å^−3^

### 

Data collection: *SMART* (Bruker, 2002 ▶); cell refinement: *SAINT* (Bruker, 2002 ▶); data reduction: *SAINT*; program(s) used to solve structure: *SHELXS97* (Sheldrick, 2008 ▶); program(s) used to refine structure: *SHELXL97* (Sheldrick, 2008 ▶); molecular graphics: *SHELXTL* (Sheldrick, 2008 ▶); software used to prepare material for publication: *SHELXTL*.

## Supplementary Material

Crystal structure: contains datablocks I, global. DOI: 10.1107/S1600536808025543/lh2667sup1.cif

Structure factors: contains datablocks I. DOI: 10.1107/S1600536808025543/lh2667Isup2.hkl

Additional supplementary materials:  crystallographic information; 3D view; checkCIF report

## Figures and Tables

**Fig. 1.**
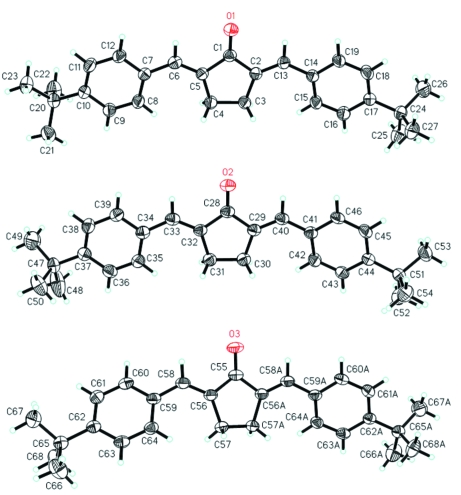
The three independent molecules of (I), showing 30% displacement ellipsoids for the non-hydrogen atoms [symmetry code: (A) -x+1, y, -z+1/2].

**Fig. 2.**
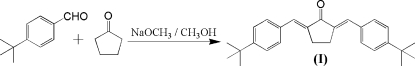
Reaction scheme.

## supplementary crystallographic information

### Comment

The title compound, (2E,5E)-2,5-bis(4-*tert*-butylbenzylidene)cyclopentanone (I), is one of the monocarbonyl analogues of curcumin designed and synthesized by our group. Curcumin has been found to possess a variety of pharmaceutical applications, for example, inhibiting the mutations and the formation of tumors, antioxidation, anti-inflammation, anti-virus, and decreasing total cholesterol and LDL cholesterol level (Began *et al.*, 1999; Kawamori *et al.*, 1999; Poorichaya *et al.*, 2007). In previous studies, curcumin was found to be able to effect the activity of enzyme 11-beta-hydroxysteroid dehydrogenase type-1 (11-beta-HSD1), which catalyzes glucocorticoid conversion between active form (corticosterone in rodents) and inactive form (dehydrocortisone in rodents) and then regulates downstream target genes leading to increased hepatic glucose production and lipolysis in adipose tissue (Livingstone & Walker, 2003; Saiah, 2008). According to the structural disadvantages of curcumin responsible for its weak pharmacokinetic profiles, a series of mono-carbonyl analogues were designed and synthesized (Liang *et al.*, 2008), and their 11-beta-HSD1-regulating activities *in vitro* were evaluated using intact Leydig cells. The title compound (I) showed an 11-beta-HSD1-regulating bioactivity with ED50 = 3029 nM on 11-beta-HSD1 oxidase and IC50 = 12278 nM against reductase of this enzyme, comparable to curcumin performs on 11-beta-HSD1 regulation (data not shown). Among these derivatives, some crystal structures are reported (Liang, Tian *et al.*, 2007; Liang, Yang *et al.*, 2007). In the present paper, we describe the crystal structure of compound (I). The geometrical parameters of title compound (I) are normal and there are three independent molecules. The dihedral angles between two benzene rings in each are 12.17 (6)°, 16.29 (11)°, 51.24 (8)°, respectively.

### Experimental

To a solution of 15 mmol 4-*tert*-butybenzaldehyde in MeOH (10 ml) was added 7.5 mmol cyclopentanone. The solution was stirred at room temperature for 20 min, followed by added dropwise 2.0 mol/*L* NaOCH_3_ (3.75 ml, 7.5 mmol) in methanol. The mixture was stirred at room temperature for 1.5 h and monitored with TLC. When the reaction was complete, the residue was poured into saturated NH_4_Cl solution and filtered. The precipitate was washed and purified by chromatography over silica gel using CH_2_Cl_2_ / CH_3_OH as the eluent to afford the pure product (yield: 66.9%). Single crystals of (I) were grown in a CH_2_Cl_2_—CH_3_CH_2_OH mixture (5:1 *v*/*v*) by slow evaporation (mp 452–454 K). 1*H*-NMR (CDCl_3_): 1.35 (18*H*, s, CH3), 3.11 (4*H*, S, CH2—CH2), 7.47 (4*H*, d, J=8.4 Hz, Ar—H(2,6)), 7.56 (4*H*, d, J=8.4 Hz, Ar—H(3,5)), 7.59 (2*H*, s, CH). ESI-MS m/*z*: 373.69 (*M*+1), calcd for C_27_H_32_O: 372.53.

### Refinement

The H atoms were positioned geometrically (C—H = 0.93–0.97 Å) and refined as riding with *U*_iso_(H) = 1.2*U*_eq_(C) or 1.5*U*_eq_(methyl C).

### Crystal data

**Table:**

C_27_H_32_O	*F*_000_ = 4040
*M**_r_* = 372.53	*D*_x_ = 1.111 Mg m^−^^3^
Monoclinic, *C*2/*c*	Mo *K*α radiation λ = 0.71073 Å
Hall symbol: -C 2yc	Cell parameters from 2156 reflections
*a* = 41.246 (7) Å	θ = 5.3–40.8º
*b* = 6.2930 (10) Å	µ = 0.07 mm^−^^1^
*c* = 43.001 (7) Å	*T* = 293 (2) K
β = 94.243 (4)º	Prism, yellow
*V* = 11131 (3) Å^3^	0.50 × 0.41 × 0.18 mm
*Z* = 20	

### Data collection

**Table:**

Bruker SMART CCD diffractometer	10373 independent reflections
Radiation source: fine-focus sealed tube	4318 reflections with *I* > 2σ(*I*)
Monochromator: graphite	*R*_int_ = 0.128
*T* = 293(2) K	θ_max_ = 25.5º
φ and ω scans	θ_min_ = 1.9º
Absorption correction: multi-scan(SADABS; Bruker, 2002)	*h* = −49→36
*T*_min_ = 0.761, *T*_max_ = 1.000	*k* = −7→7
28046 measured reflections	*l* = −48→52

### Refinement

**Table:**

Refinement on *F*^2^	Hydrogen site location: inferred from neighbouring sites
Least-squares matrix: full	H-atom parameters constrained
*R*[*F*^2^ > 2σ(*F*^2^)] = 0.068	*w* = 1/[σ^2^(*F*_o_^2^) + (0.0573*P*)^2^] where *P* = (*F*_o_^2^ + 2*F*_c_^2^)/3
*wR*(*F*^2^) = 0.180	(Δ/σ)_max_ = 0.011
*S* = 0.86	Δρ_max_ = 0.20 e Å^−^^3^
10373 reflections	Δρ_min_ = −0.18 e Å^−^^3^
648 parameters	Extinction correction: SHELXL97 (Sheldrick, 2008), Fc^*^=kFc[1+0.001xFc^2^λ^3^/sin(2θ)]^-1/4^
Primary atom site location: structure-invariant direct methods	Extinction coefficient: 0.00016 (4)
Secondary atom site location: difference Fourier map	

### Special details

**Table:**

Geometry. All e.s.d.'s (except the e.s.d. in the dihedral angle between two l.s. planes) are estimated using the full covariance matrix. The cell e.s.d.'s are taken into account individually in the estimation of e.s.d.'s in distances, angles and torsion angles; correlations between e.s.d.'s in cell parameters are only used when they are defined by crystal symmetry. An approximate (isotropic) treatment of cell e.s.d.'s is used for estimating e.s.d.'s involving l.s. planes.
Refinement. Refinement of *F*^2^ against ALL reflections. The weighted *R*-factor *wR* and goodness of fit *S* are based on *F*^2^, conventional *R*-factors *R* are based on *F*, with *F* set to zero for negative *F*^2^. The threshold expression of *F*^2^ > σ(*F*^2^) is used only for calculating *R*-factors(gt) *etc*. and is not relevant to the choice of reflections for refinement. *R*-factors based on *F*^2^ are statistically about twice as large as those based on *F*, and *R*- factors based on ALL data will be even larger.

### Fractional atomic coordinates and isotropic or equivalent isotropic displacement parameters (Å^2^)

**Table:**

	*x*	*y*	*z*	*U*_iso_*/*U*_eq_	
O1	0.29393 (5)	0.4048 (3)	0.07533 (6)	0.0780 (7)	
O2	0.39495 (5)	0.5016 (3)	0.17171 (6)	0.0781 (7)	
O3	0.5000	0.4826 (5)	0.2500	0.150 (2)	
C1	0.29711 (7)	0.5803 (5)	0.06428 (7)	0.0541 (8)	
C2	0.27036 (7)	0.7186 (5)	0.05130 (7)	0.0518 (8)	
C3	0.28460 (7)	0.9189 (5)	0.03993 (7)	0.0564 (8)	
H3A	0.2770	1.0396	0.0514	0.068*	
H3B	0.2782	0.9391	0.0180	0.068*	
C4	0.32163 (7)	0.9001 (5)	0.04504 (7)	0.0609 (9)	
H4A	0.3314	0.9010	0.0252	0.073*	
H4B	0.3305	1.0176	0.0576	0.073*	
C5	0.32819 (7)	0.6937 (4)	0.06157 (7)	0.0498 (8)	
C6	0.35604 (7)	0.6012 (5)	0.07231 (7)	0.0560 (8)	
H6	0.3533	0.4710	0.0820	0.067*	
C7	0.38955 (7)	0.6671 (5)	0.07152 (7)	0.0504 (8)	
C8	0.39985 (8)	0.8547 (5)	0.05871 (8)	0.0693 (10)	
H8	0.3844	0.9530	0.0512	0.083*	
C9	0.43234 (8)	0.9011 (5)	0.05671 (7)	0.0662 (10)	
H9	0.4380	1.0308	0.0483	0.079*	
C10	0.45680 (7)	0.7603 (5)	0.06689 (7)	0.0501 (8)	
C11	0.44633 (8)	0.5780 (5)	0.08082 (8)	0.0676 (10)	
H11	0.4618	0.4827	0.0893	0.081*	
C12	0.41393 (8)	0.5305 (5)	0.08281 (8)	0.0669 (10)	
H12	0.4083	0.4032	0.0920	0.080*	
C13	0.23963 (7)	0.6558 (5)	0.05233 (7)	0.0569 (9)	
H13	0.2373	0.5182	0.0597	0.068*	
C14	0.20898 (7)	0.7645 (5)	0.04394 (7)	0.0521 (8)	
C15	0.20580 (8)	0.9549 (5)	0.02837 (8)	0.0651 (9)	
H15	0.2242	1.0205	0.0216	0.078*	
C16	0.17589 (8)	1.0512 (5)	0.02251 (8)	0.0691 (10)	
H16	0.1746	1.1799	0.0119	0.083*	
C17	0.14765 (7)	0.9602 (5)	0.03214 (7)	0.0516 (8)	
C18	0.15075 (8)	0.7686 (5)	0.04666 (8)	0.0693 (10)	
H18	0.1322	0.7016	0.0530	0.083*	
C19	0.18059 (8)	0.6705 (5)	0.05234 (8)	0.0683 (10)	
H19	0.1816	0.5382	0.0620	0.082*	
C20	0.49193 (7)	0.8047 (5)	0.06146 (7)	0.0586 (9)	
C21	0.50018 (8)	1.0402 (6)	0.06386 (8)	0.0873 (12)	
H21A	0.4866	1.1179	0.0487	0.121*	
H21B	0.5226	1.0609	0.0600	0.121*	
H21C	0.4965	1.0905	0.0844	0.121*	
C22	0.49753 (8)	0.7297 (6)	0.02807 (7)	0.0924 (13)	
H22A	0.4935	0.5797	0.0264	0.129*	
H22B	0.5196	0.7589	0.0237	0.129*	
H22C	0.4830	0.8039	0.0134	0.129*	
C23	0.51543 (7)	0.6833 (5)	0.08448 (8)	0.0813 (11)	
H23A	0.5115	0.7228	0.1054	0.112*	
H23B	0.5374	0.7180	0.0806	0.112*	
H23C	0.5121	0.5333	0.0818	0.112*	
C24	0.11478 (7)	1.0750 (5)	0.02828 (7)	0.0600 (9)	
C25	0.11316 (9)	1.2364 (6)	0.00167 (8)	0.0994 (13)	
H25A	0.0917	1.2959	−0.0009	0.129*	
H25B	0.1181	1.1670	−0.0173	0.129*	
H25C	0.1286	1.3477	0.0065	0.129*	
C26	0.08709 (8)	0.9174 (6)	0.02227 (10)	0.1145 (16)	
H26A	0.0856	0.8292	0.0403	0.132*	
H26B	0.0912	0.8303	0.0046	0.132*	
H26C	0.0671	0.9933	0.0180	0.132*	
C27	0.10989 (8)	1.1952 (5)	0.05843 (8)	0.0823 (11)	
H27A	0.1092	1.0959	0.0753	0.113*	
H27B	0.0898	1.2729	0.0562	0.113*	
H27C	0.1276	1.2922	0.0628	0.113*	
C28	0.39418 (8)	0.6813 (5)	0.16108 (7)	0.0569 (9)	
C29	0.36451 (8)	0.8006 (5)	0.14964 (7)	0.0530 (8)	
C30	0.37431 (7)	1.0141 (5)	0.13873 (7)	0.0610 (9)	
H30A	0.3645	1.1246	0.1507	0.073*	
H30B	0.3673	1.0331	0.1169	0.073*	
C31	0.41147 (7)	1.0257 (5)	0.14342 (7)	0.0598 (9)	
H31A	0.4208	1.0478	0.1236	0.072*	
H31B	0.4180	1.1422	0.1572	0.072*	
C32	0.42270 (8)	0.8190 (5)	0.15740 (7)	0.0525 (8)	
C33	0.45271 (8)	0.7498 (5)	0.16590 (7)	0.0591 (9)	
H33	0.4536	0.6132	0.1742	0.071*	
C34	0.48430 (8)	0.8524 (5)	0.16440 (7)	0.0572 (9)	
C35	0.48909 (8)	1.0564 (5)	0.15346 (8)	0.0651 (9)	
H35	0.4712	1.1402	0.1474	0.078*	
C36	0.51997 (9)	1.1379 (5)	0.15140 (8)	0.0693 (10)	
H36	0.5221	1.2766	0.1444	0.083*	
C37	0.54771 (8)	1.0213 (6)	0.15933 (7)	0.0596 (9)	
C38	0.54279 (8)	0.8225 (6)	0.17108 (8)	0.0700 (10)	
H38	0.5608	0.7416	0.1778	0.084*	
C39	0.51227 (8)	0.7368 (5)	0.17340 (7)	0.0656 (9)	
H39	0.5103	0.5996	0.1811	0.079*	
C40	0.33520 (8)	0.7134 (5)	0.15129 (7)	0.0575 (9)	
H40	0.3354	0.5726	0.1579	0.069*	
C41	0.30296 (8)	0.8035 (5)	0.14442 (7)	0.0542 (8)	
C42	0.29618 (8)	0.9955 (5)	0.13020 (7)	0.0650 (9)	
H42	0.3131	1.0715	0.1223	0.078*	
C43	0.26539 (8)	1.0797 (5)	0.12713 (7)	0.0621 (9)	
H43	0.2621	1.2104	0.1173	0.075*	
C44	0.23909 (7)	0.9745 (5)	0.13833 (6)	0.0487 (8)	
C45	0.24561 (8)	0.7785 (5)	0.15148 (8)	0.0692 (10)	
H45	0.2286	0.7013	0.1589	0.083*	
C46	0.27610 (8)	0.6926 (5)	0.15411 (8)	0.0697 (10)	
H46	0.2791	0.5573	0.1625	0.084*	
C47	0.58159 (8)	1.1126 (6)	0.15588 (8)	0.0694 (10)	
C48	0.58196 (9)	1.2481 (8)	0.12684 (10)	0.116 (2)	
H48A	0.5704	1.3778	0.1298	0.133*	
H48B	0.5717	1.1721	0.1094	0.133*	
H48C	0.6040	1.2803	0.1229	0.133*	
C49	0.60698 (9)	0.9422 (7)	0.15397 (13)	0.115 (2)	
H49A	0.6092	0.8657	0.1733	0.132*	
H49B	0.6274	1.0063	0.1500	0.132*	
H49C	0.6005	0.8460	0.1373	0.132*	
C50	0.59097 (9)	1.2498 (6)	0.18397 (10)	0.1174 (16)	
H50A	0.5930	1.1627	0.2023	0.136*	
H50B	0.5745	1.3556	0.1861	0.136*	
H50C	0.6114	1.3181	0.1813	0.136*	
C51	0.20573 (7)	1.0771 (5)	0.13858 (7)	0.0539 (8)	
C52	0.19933 (8)	1.2366 (5)	0.11190 (8)	0.0842 (11)	
H52A	0.2155	1.3465	0.1136	0.116*	
H52B	0.1782	1.2984	0.1131	0.116*	
H52C	0.2003	1.1649	0.0923	0.116*	
C53	0.17837 (8)	0.9136 (6)	0.13635 (9)	0.0906 (12)	
H53A	0.1798	0.8295	0.1179	0.126*	
H53B	0.1578	0.9854	0.1354	0.126*	
H53C	0.1803	0.8230	0.1543	0.126*	
C54	0.20421 (8)	1.1991 (5)	0.16945 (8)	0.0819 (11)	
H54A	0.2071	1.1016	0.1866	0.113*	
H54B	0.1835	1.2676	0.1699	0.113*	
H54C	0.2211	1.3041	0.1711	0.113*	
C55	0.5000	0.6744 (8)	0.2500	0.0757 (15)	
C56	0.47090 (8)	0.8110 (5)	0.24588 (7)	0.0558 (8)	
C57	0.48172 (6)	1.0344 (4)	0.24519 (7)	0.0578 (9)	
H57A	0.4701	1.1193	0.2596	0.069*	
H57B	0.4776	1.0929	0.2244	0.069*	
C58	0.44126 (8)	0.7242 (5)	0.24475 (7)	0.0621 (9)	
H58	0.4408	0.5766	0.2457	0.075*	
C59	0.40947 (8)	0.8272 (5)	0.24219 (7)	0.0541 (8)	
C60	0.38304 (8)	0.7181 (5)	0.25263 (8)	0.0692 (10)	
H60	0.3861	0.5811	0.2604	0.083*	
C61	0.35248 (8)	0.8073 (5)	0.25171 (8)	0.0694 (10)	
H61	0.3356	0.7305	0.2595	0.083*	
C62	0.34621 (7)	1.0063 (5)	0.23968 (7)	0.0508 (8)	
C63	0.37253 (8)	1.1119 (5)	0.22831 (7)	0.0576 (9)	
H63	0.3692	1.2456	0.2194	0.069*	
C64	0.40330 (8)	1.0253 (5)	0.22976 (7)	0.0570 (9)	
H64	0.4202	1.1027	0.2222	0.068*	
C65	0.31284 (8)	1.1083 (5)	0.24011 (7)	0.0554 (8)	
C66	0.30633 (8)	1.2711 (6)	0.21395 (8)	0.0984 (13)	
H66A	0.3220	1.3839	0.2164	0.128*	
H66B	0.2848	1.3282	0.2148	0.128*	
H66C	0.3081	1.2033	0.1942	0.128*	
C67	0.28577 (8)	0.9443 (6)	0.23752 (10)	0.1012 (14)	
H67A	0.2864	0.8687	0.2182	0.132*	
H67B	0.2652	1.0144	0.2381	0.132*	
H67C	0.2886	0.8461	0.2546	0.132*	
C68	0.31128 (8)	1.2240 (6)	0.27122 (8)	0.0887 (12)	
H68A	0.3141	1.1236	0.2880	0.123*	
H68B	0.2905	1.2922	0.2719	0.123*	
H68C	0.3282	1.3289	0.2733	0.123*	

### Atomic displacement parameters (Å^2^)

**Table:**

	*U*^11^	*U*^22^	*U*^33^	*U*^12^	*U*^13^	*U*^23^
O1	0.0684 (16)	0.0598 (15)	0.107 (2)	−0.0006 (13)	0.0149 (13)	0.0237 (15)
O2	0.0815 (17)	0.0480 (14)	0.106 (2)	0.0022 (13)	0.0130 (14)	0.0113 (14)
O3	0.081 (3)	0.036 (2)	0.332 (7)	0.000	0.016 (3)	0.000
C1	0.059 (2)	0.050 (2)	0.055 (2)	0.0021 (18)	0.0105 (16)	0.0025 (17)
C2	0.054 (2)	0.053 (2)	0.049 (2)	0.0001 (18)	0.0108 (15)	−0.0013 (16)
C3	0.054 (2)	0.058 (2)	0.058 (2)	−0.0018 (17)	0.0104 (16)	0.0071 (16)
C4	0.057 (2)	0.062 (2)	0.064 (2)	0.0006 (17)	0.0066 (16)	0.0128 (18)
C5	0.051 (2)	0.051 (2)	0.0484 (19)	0.0035 (17)	0.0133 (15)	0.0062 (15)
C6	0.064 (2)	0.057 (2)	0.048 (2)	0.0030 (18)	0.0105 (16)	0.0040 (16)
C7	0.054 (2)	0.053 (2)	0.0443 (19)	0.0046 (17)	0.0059 (15)	0.0060 (15)
C8	0.053 (2)	0.069 (2)	0.086 (3)	0.0052 (19)	0.0015 (18)	0.022 (2)
C9	0.057 (2)	0.066 (2)	0.076 (3)	−0.0001 (19)	0.0036 (18)	0.0237 (19)
C10	0.052 (2)	0.059 (2)	0.0396 (18)	0.0071 (17)	0.0036 (14)	−0.0024 (16)
C11	0.056 (2)	0.070 (2)	0.077 (3)	0.0150 (19)	0.0045 (18)	0.017 (2)
C12	0.059 (2)	0.060 (2)	0.082 (3)	0.0062 (19)	0.0071 (19)	0.0227 (19)
C13	0.055 (2)	0.055 (2)	0.062 (2)	0.0012 (18)	0.0118 (16)	0.0019 (16)
C14	0.054 (2)	0.053 (2)	0.049 (2)	−0.0008 (17)	0.0067 (15)	0.0050 (16)
C15	0.053 (2)	0.070 (2)	0.075 (3)	−0.0006 (19)	0.0177 (17)	0.0171 (19)
C16	0.063 (2)	0.070 (2)	0.075 (3)	0.005 (2)	0.0118 (19)	0.0223 (19)
C17	0.054 (2)	0.056 (2)	0.0454 (19)	0.0002 (17)	0.0033 (15)	0.0007 (16)
C18	0.055 (2)	0.076 (3)	0.077 (3)	−0.0073 (19)	0.0105 (18)	0.024 (2)
C19	0.055 (2)	0.064 (2)	0.086 (3)	−0.0021 (19)	0.0052 (19)	0.0267 (19)
C20	0.053 (2)	0.075 (2)	0.048 (2)	0.0003 (19)	0.0033 (15)	−0.0044 (18)
C21	0.068 (3)	0.098 (3)	0.097 (3)	−0.013 (2)	0.012 (2)	−0.006 (2)
C22	0.072 (3)	0.148 (4)	0.060 (3)	0.001 (2)	0.0227 (19)	−0.015 (2)
C23	0.058 (2)	0.114 (3)	0.072 (3)	0.012 (2)	0.0023 (18)	0.010 (2)
C24	0.058 (2)	0.067 (2)	0.055 (2)	0.0061 (18)	−0.0008 (16)	0.0030 (18)
C25	0.099 (3)	0.126 (3)	0.073 (3)	0.041 (3)	0.006 (2)	0.031 (2)
C26	0.062 (3)	0.101 (3)	0.175 (5)	−0.005 (2)	−0.030 (3)	−0.027 (3)
C27	0.074 (3)	0.100 (3)	0.074 (3)	0.014 (2)	0.012 (2)	−0.008 (2)
C28	0.069 (2)	0.049 (2)	0.055 (2)	0.0024 (19)	0.0134 (17)	−0.0054 (17)
C29	0.064 (2)	0.048 (2)	0.048 (2)	0.0015 (18)	0.0161 (16)	−0.0042 (15)
C30	0.067 (2)	0.059 (2)	0.058 (2)	0.0008 (18)	0.0115 (17)	0.0045 (17)
C31	0.062 (2)	0.059 (2)	0.059 (2)	−0.0003 (18)	0.0093 (16)	0.0041 (17)
C32	0.061 (2)	0.046 (2)	0.052 (2)	0.0036 (18)	0.0117 (16)	−0.0029 (16)
C33	0.073 (2)	0.052 (2)	0.054 (2)	0.0012 (19)	0.0167 (17)	−0.0035 (16)
C34	0.065 (2)	0.058 (2)	0.049 (2)	0.0037 (19)	0.0109 (16)	−0.0072 (17)
C35	0.060 (2)	0.061 (2)	0.075 (3)	0.0040 (19)	0.0121 (18)	−0.0022 (19)
C36	0.075 (3)	0.061 (2)	0.074 (3)	−0.006 (2)	0.015 (2)	0.0010 (19)
C37	0.065 (2)	0.062 (2)	0.052 (2)	−0.001 (2)	0.0042 (17)	−0.0049 (17)
C38	0.058 (2)	0.079 (3)	0.072 (3)	0.005 (2)	−0.0030 (18)	0.004 (2)
C39	0.065 (2)	0.061 (2)	0.071 (3)	0.006 (2)	0.0033 (18)	0.0053 (18)
C40	0.069 (2)	0.049 (2)	0.056 (2)	−0.0004 (18)	0.0148 (17)	0.0010 (16)
C41	0.064 (2)	0.051 (2)	0.049 (2)	−0.0018 (18)	0.0099 (16)	0.0044 (16)
C42	0.060 (2)	0.069 (2)	0.068 (2)	−0.0015 (19)	0.0192 (17)	0.0224 (19)
C43	0.069 (2)	0.055 (2)	0.064 (2)	−0.0048 (19)	0.0135 (18)	0.0155 (17)
C44	0.059 (2)	0.051 (2)	0.0366 (18)	−0.0042 (17)	0.0027 (14)	0.0015 (15)
C45	0.063 (2)	0.063 (2)	0.082 (3)	−0.0110 (19)	0.0137 (19)	0.021 (2)
C46	0.068 (3)	0.050 (2)	0.091 (3)	−0.007 (2)	0.004 (2)	0.0208 (19)
C47	0.055 (2)	0.081 (3)	0.071 (3)	−0.008 (2)	−0.0033 (18)	−0.004 (2)
C48	0.093 (3)	0.177 (6)	0.095 (4)	−0.069 (4)	−0.007 (3)	0.087 (4)
C49	0.070 (3)	0.120 (4)	0.180 (7)	0.003 (3)	0.057 (4)	−0.020 (4)
C50	0.101 (3)	0.139 (4)	0.109 (4)	−0.046 (3)	−0.010 (3)	−0.020 (3)
C51	0.058 (2)	0.058 (2)	0.046 (2)	−0.0061 (17)	0.0041 (15)	0.0049 (16)
C52	0.083 (3)	0.104 (3)	0.065 (3)	0.017 (2)	0.0042 (19)	0.029 (2)
C53	0.066 (3)	0.096 (3)	0.109 (3)	−0.017 (2)	−0.002 (2)	0.005 (2)
C54	0.086 (3)	0.093 (3)	0.068 (3)	0.014 (2)	0.010 (2)	−0.005 (2)
C55	0.076 (4)	0.038 (3)	0.114 (5)	0.000	0.017 (3)	0.000
C56	0.065 (2)	0.0397 (19)	0.063 (2)	−0.0012 (17)	0.0130 (17)	−0.0012 (16)
C57	0.063 (2)	0.0414 (19)	0.070 (2)	−0.0032 (15)	0.0114 (18)	0.0011 (16)
C58	0.073 (3)	0.0409 (19)	0.073 (2)	0.0005 (19)	0.0110 (18)	−0.0013 (17)
C59	0.070 (2)	0.0397 (19)	0.054 (2)	−0.0034 (17)	0.0093 (17)	−0.0054 (16)
C60	0.075 (3)	0.044 (2)	0.090 (3)	−0.006 (2)	0.013 (2)	0.0126 (18)
C61	0.066 (3)	0.059 (2)	0.085 (3)	−0.0039 (19)	0.0184 (19)	0.015 (2)
C62	0.064 (2)	0.047 (2)	0.0414 (19)	−0.0034 (17)	0.0048 (15)	−0.0054 (15)
C63	0.078 (3)	0.047 (2)	0.048 (2)	−0.0015 (19)	0.0051 (17)	0.0064 (16)
C64	0.068 (2)	0.050 (2)	0.055 (2)	−0.0060 (18)	0.0155 (17)	0.0035 (16)
C65	0.064 (2)	0.057 (2)	0.045 (2)	−0.0012 (18)	0.0024 (15)	−0.0043 (16)
C66	0.101 (3)	0.122 (3)	0.071 (3)	0.033 (3)	0.004 (2)	0.027 (2)
C67	0.069 (3)	0.091 (3)	0.142 (4)	−0.006 (2)	−0.006 (2)	−0.011 (3)
C68	0.085 (3)	0.112 (3)	0.070 (3)	0.021 (2)	0.011 (2)	−0.021 (2)

### Geometric parameters (Å, °)

**Table:**

O1—C1	1.213 (3)	C35—C36	1.382 (4)
O2—C28	1.219 (3)	C35—H35	0.9300
O3—C55	1.207 (5)	C36—C37	1.381 (4)
C1—C5	1.479 (4)	C36—H36	0.9300
C1—C2	1.482 (4)	C37—C38	1.370 (4)
C2—C13	1.331 (4)	C37—C47	1.528 (4)
C2—C3	1.489 (4)	C38—C39	1.380 (4)
C3—C4	1.532 (3)	C38—H38	0.9300
C3—H3A	0.9700	C39—H39	0.9300
C3—H3B	0.9700	C40—C41	1.456 (4)
C4—C5	1.496 (4)	C40—H40	0.9300
C4—H4A	0.9700	C41—C42	1.374 (4)
C4—H4B	0.9700	C41—C46	1.399 (4)
C5—C6	1.339 (4)	C42—C43	1.374 (4)
C6—C7	1.446 (4)	C42—H42	0.9300
C6—H6	0.9300	C43—C44	1.387 (4)
C7—C12	1.383 (4)	C43—H43	0.9300
C7—C8	1.383 (4)	C44—C45	1.375 (4)
C8—C9	1.381 (4)	C44—C51	1.521 (4)
C8—H8	0.9300	C45—C46	1.366 (4)
C9—C10	1.389 (4)	C45—H45	0.9300
C9—H9	0.9300	C46—H46	0.9300
C10—C11	1.379 (4)	C47—C49	1.505 (5)
C10—C20	1.511 (4)	C47—C50	1.512 (4)
C11—C12	1.378 (4)	C47—C48	1.513 (4)
C11—H11	0.9300	C48—H48A	0.9600
C12—H12	0.9300	C48—H48B	0.9600
C13—C14	1.459 (4)	C48—H48C	0.9600
C13—H13	0.9300	C49—H49A	0.9600
C14—C15	1.374 (4)	C49—H49B	0.9600
C14—C19	1.384 (4)	C49—H49C	0.9600
C15—C16	1.381 (4)	C50—H50A	0.9600
C15—H15	0.9300	C50—H50B	0.9600
C16—C17	1.388 (4)	C50—H50C	0.9600
C16—H16	0.9300	C51—C53	1.524 (4)
C17—C18	1.359 (4)	C51—C52	1.533 (4)
C17—C24	1.534 (4)	C51—C54	1.539 (4)
C18—C19	1.383 (4)	C52—H52A	0.9600
C18—H18	0.9300	C52—H52B	0.9600
C19—H19	0.9300	C52—H52C	0.9600
C20—C21	1.523 (4)	C53—H53A	0.9600
C20—C23	1.536 (4)	C53—H53B	0.9600
C20—C22	1.545 (4)	C53—H53C	0.9600
C21—H21A	0.9600	C54—H54A	0.9600
C21—H21B	0.9600	C54—H54B	0.9600
C21—H21C	0.9600	C54—H54C	0.9600
C22—H22A	0.9600	C55—C56^i^	1.476 (4)
C22—H22B	0.9600	C55—C56	1.476 (4)
C22—H22C	0.9600	C56—C58	1.337 (4)
C23—H23A	0.9600	C56—C57	1.476 (4)
C23—H23B	0.9600	C57—C57^i^	1.534 (5)
C23—H23C	0.9600	C57—H57A	0.9700
C24—C26	1.520 (4)	C57—H57B	0.9700
C24—C27	1.527 (4)	C58—C59	1.460 (4)
C24—C25	1.528 (4)	C58—H58	0.9300
C25—H25A	0.9600	C59—C64	1.373 (4)
C25—H25B	0.9600	C59—C60	1.391 (4)
C25—H25C	0.9600	C60—C61	1.378 (4)
C26—H26A	0.9600	C60—H60	0.9300
C26—H26B	0.9600	C61—C62	1.372 (4)
C26—H26C	0.9600	C61—H61	0.9300
C27—H27A	0.9600	C62—C63	1.392 (4)
C27—H27B	0.9600	C62—C65	1.520 (4)
C27—H27C	0.9600	C63—C64	1.379 (4)
C28—C32	1.479 (4)	C63—H63	0.9300
C28—C29	1.488 (4)	C64—H64	0.9300
C29—C40	1.334 (4)	C65—C67	1.518 (4)
C29—C30	1.489 (4)	C65—C68	1.529 (4)
C30—C31	1.532 (4)	C65—C66	1.530 (4)
C30—H30A	0.9700	C66—H66A	0.9600
C30—H30B	0.9700	C66—H66B	0.9600
C31—C32	1.492 (4)	C66—H66C	0.9600
C31—H31A	0.9700	C67—H67A	0.9600
C31—H31B	0.9700	C67—H67B	0.9600
C32—C33	1.337 (4)	C67—H67C	0.9600
C33—C34	1.460 (4)	C68—H68A	0.9600
C33—H33	0.9300	C68—H68B	0.9600
C34—C35	1.387 (4)	C68—H68C	0.9600
C34—C39	1.394 (4)		
			
O1—C1—C5	126.1 (3)	C34—C35—H35	119.3
O1—C1—C2	125.7 (3)	C37—C36—C35	122.5 (3)
C5—C1—C2	108.2 (3)	C37—C36—H36	118.7
C13—C2—C1	119.8 (3)	C35—C36—H36	118.7
C13—C2—C3	131.5 (3)	C38—C37—C36	115.7 (3)
C1—C2—C3	108.7 (3)	C38—C37—C47	122.7 (3)
C2—C3—C4	107.5 (2)	C36—C37—C47	121.6 (3)
C2—C3—H3A	110.2	C37—C38—C39	123.0 (3)
C4—C3—H3A	110.2	C37—C38—H38	118.5
C2—C3—H3B	110.2	C39—C38—H38	118.5
C4—C3—H3B	110.2	C38—C39—C34	121.1 (3)
H3A—C3—H3B	108.5	C38—C39—H39	119.4
C5—C4—C3	106.2 (2)	C34—C39—H39	119.4
C5—C4—H4A	110.5	C29—C40—C41	130.3 (3)
C3—C4—H4A	110.5	C29—C40—H40	114.9
C5—C4—H4B	110.5	C41—C40—H40	114.9
C3—C4—H4B	110.5	C42—C41—C46	115.6 (3)
H4A—C4—H4B	108.7	C42—C41—C40	125.9 (3)
C6—C5—C1	119.3 (3)	C46—C41—C40	118.5 (3)
C6—C5—C4	131.4 (3)	C43—C42—C41	122.6 (3)
C1—C5—C4	109.2 (3)	C43—C42—H42	118.7
C5—C6—C7	131.6 (3)	C41—C42—H42	118.7
C5—C6—H6	114.2	C42—C43—C44	121.6 (3)
C7—C6—H6	114.2	C42—C43—H43	119.2
C12—C7—C8	115.7 (3)	C44—C43—H43	119.2
C12—C7—C6	119.0 (3)	C45—C44—C43	115.9 (3)
C8—C7—C6	125.2 (3)	C45—C44—C51	121.8 (3)
C9—C8—C7	122.3 (3)	C43—C44—C51	122.1 (3)
C9—C8—H8	118.9	C46—C45—C44	122.7 (3)
C7—C8—H8	118.9	C46—C45—H45	118.7
C8—C9—C10	122.1 (3)	C44—C45—H45	118.7
C8—C9—H9	119.0	C45—C46—C41	121.5 (3)
C10—C9—H9	119.0	C45—C46—H46	119.2
C11—C10—C9	115.1 (3)	C41—C46—H46	119.2
C11—C10—C20	123.8 (3)	C49—C47—C50	108.2 (3)
C9—C10—C20	121.1 (3)	C49—C47—C48	107.9 (4)
C12—C11—C10	122.9 (3)	C50—C47—C48	108.7 (3)
C12—C11—H11	118.5	C49—C47—C37	112.5 (3)
C10—C11—H11	118.5	C50—C47—C37	108.6 (3)
C11—C12—C7	121.8 (3)	C48—C47—C37	110.9 (3)
C11—C12—H12	119.1	C47—C48—H48A	109.5
C7—C12—H12	119.1	C47—C48—H48B	109.5
C2—C13—C14	131.5 (3)	H48A—C48—H48B	109.5
C2—C13—H13	114.3	C47—C48—H48C	109.5
C14—C13—H13	114.3	H48A—C48—H48C	109.5
C15—C14—C19	116.6 (3)	H48B—C48—H48C	109.5
C15—C14—C13	125.4 (3)	C47—C49—H49A	109.5
C19—C14—C13	118.0 (3)	C47—C49—H49B	109.5
C14—C15—C16	121.6 (3)	H49A—C49—H49B	109.5
C14—C15—H15	119.2	C47—C49—H49C	109.5
C16—C15—H15	119.2	H49A—C49—H49C	109.5
C15—C16—C17	121.5 (3)	H49B—C49—H49C	109.5
C15—C16—H16	119.3	C47—C50—H50A	109.5
C17—C16—H16	119.3	C47—C50—H50B	109.5
C18—C17—C16	116.8 (3)	H50A—C50—H50B	109.5
C18—C17—C24	121.3 (3)	C47—C50—H50C	109.5
C16—C17—C24	121.8 (3)	H50A—C50—H50C	109.5
C17—C18—C19	122.0 (3)	H50B—C50—H50C	109.5
C17—C18—H18	119.0	C44—C51—C53	112.3 (3)
C19—C18—H18	119.0	C44—C51—C52	112.2 (3)
C18—C19—C14	121.5 (3)	C53—C51—C52	108.0 (3)
C18—C19—H19	119.3	C44—C51—C54	108.2 (2)
C14—C19—H19	119.3	C53—C51—C54	108.3 (3)
C10—C20—C21	112.4 (3)	C52—C51—C54	107.6 (3)
C10—C20—C23	112.0 (3)	C51—C52—H52A	109.5
C21—C20—C23	108.2 (3)	C51—C52—H52B	109.5
C10—C20—C22	107.4 (2)	H52A—C52—H52B	109.5
C21—C20—C22	108.2 (3)	C51—C52—H52C	109.5
C23—C20—C22	108.4 (3)	H52A—C52—H52C	109.5
C20—C21—H21A	109.5	H52B—C52—H52C	109.5
C20—C21—H21B	109.5	C51—C53—H53A	109.5
H21A—C21—H21B	109.5	C51—C53—H53B	109.5
C20—C21—H21C	109.5	H53A—C53—H53B	109.5
H21A—C21—H21C	109.5	C51—C53—H53C	109.5
H21B—C21—H21C	109.5	H53A—C53—H53C	109.5
C20—C22—H22A	109.5	H53B—C53—H53C	109.5
C20—C22—H22B	109.5	C51—C54—H54A	109.5
H22A—C22—H22B	109.5	C51—C54—H54B	109.5
C20—C22—H22C	109.5	H54A—C54—H54B	109.5
H22A—C22—H22C	109.5	C51—C54—H54C	109.5
H22B—C22—H22C	109.5	H54A—C54—H54C	109.5
C20—C23—H23A	109.5	H54B—C54—H54C	109.5
C20—C23—H23B	109.5	O3—C55—C56^i^	125.6 (2)
H23A—C23—H23B	109.5	O3—C55—C56	125.6 (2)
C20—C23—H23C	109.5	C56^i^—C55—C56	108.8 (4)
H23A—C23—H23C	109.5	C58—C56—C55	120.0 (3)
H23B—C23—H23C	109.5	C58—C56—C57	131.7 (3)
C26—C24—C27	108.8 (3)	C55—C56—C57	108.2 (3)
C26—C24—C25	108.4 (3)	C56—C57—C57^i^	106.69 (17)
C27—C24—C25	107.8 (3)	C56—C57—H57A	110.4
C26—C24—C17	111.0 (3)	C57^i^—C57—H57A	110.4
C27—C24—C17	108.2 (2)	C56—C57—H57B	110.4
C25—C24—C17	112.5 (3)	C57^i^—C57—H57B	110.4
C24—C25—H25A	109.5	H57A—C57—H57B	108.6
C24—C25—H25B	109.5	C56—C58—C59	129.4 (3)
H25A—C25—H25B	109.5	C56—C58—H58	115.3
C24—C25—H25C	109.5	C59—C58—H58	115.3
H25A—C25—H25C	109.5	C64—C59—C60	116.6 (3)
H25B—C25—H25C	109.5	C64—C59—C58	124.9 (3)
C24—C26—H26A	109.5	C60—C59—C58	118.5 (3)
C24—C26—H26B	109.5	C61—C60—C59	121.8 (3)
H26A—C26—H26B	109.5	C61—C60—H60	119.1
C24—C26—H26C	109.5	C59—C60—H60	119.1
H26A—C26—H26C	109.5	C62—C61—C60	122.0 (3)
H26B—C26—H26C	109.5	C62—C61—H61	119.0
C24—C27—H27A	109.5	C60—C61—H61	119.0
C24—C27—H27B	109.5	C61—C62—C63	116.0 (3)
H27A—C27—H27B	109.5	C61—C62—C65	121.8 (3)
C24—C27—H27C	109.5	C63—C62—C65	122.2 (3)
H27A—C27—H27C	109.5	C64—C63—C62	122.3 (3)
H27B—C27—H27C	109.5	C64—C63—H63	118.8
O2—C28—C32	125.8 (3)	C62—C63—H63	118.8
O2—C28—C29	126.2 (3)	C59—C64—C63	121.3 (3)
C32—C28—C29	108.1 (3)	C59—C64—H64	119.3
C40—C29—C28	120.1 (3)	C63—C64—H64	119.3
C40—C29—C30	131.0 (3)	C67—C65—C62	111.9 (3)
C28—C29—C30	108.9 (3)	C67—C65—C68	108.0 (3)
C29—C30—C31	107.1 (2)	C62—C65—C68	108.0 (2)
C29—C30—H30A	110.3	C67—C65—C66	108.2 (3)
C31—C30—H30A	110.3	C62—C65—C66	112.6 (3)
C29—C30—H30B	110.3	C68—C65—C66	107.9 (3)
C31—C30—H30B	110.3	C65—C66—H66A	109.5
H30A—C30—H30B	108.6	C65—C66—H66B	109.5
C32—C31—C30	106.8 (2)	H66A—C66—H66B	109.5
C32—C31—H31A	110.4	C65—C66—H66C	109.5
C30—C31—H31A	110.4	H66A—C66—H66C	109.5
C32—C31—H31B	110.4	H66B—C66—H66C	109.5
C30—C31—H31B	110.4	C65—C67—H67A	109.5
H31A—C31—H31B	108.6	C65—C67—H67B	109.5
C33—C32—C28	120.5 (3)	H67A—C67—H67B	109.5
C33—C32—C31	130.4 (3)	C65—C67—H67C	109.5
C28—C32—C31	109.1 (3)	H67A—C67—H67C	109.5
C32—C33—C34	130.9 (3)	H67B—C67—H67C	109.5
C32—C33—H33	114.5	C65—C68—H68A	109.5
C34—C33—H33	114.5	C65—C68—H68B	109.5
C35—C34—C39	116.2 (3)	H68A—C68—H68B	109.5
C35—C34—C33	125.1 (3)	C65—C68—H68C	109.5
C39—C34—C33	118.7 (3)	H68A—C68—H68C	109.5
C36—C35—C34	121.4 (3)	H68B—C68—H68C	109.5
C36—C35—H35	119.3		
			
O1—C1—C2—C13	2.8 (5)	C28—C32—C33—C34	179.3 (3)
C5—C1—C2—C13	−177.0 (3)	C31—C32—C33—C34	0.0 (5)
O1—C1—C2—C3	−179.5 (3)	C32—C33—C34—C35	1.1 (5)
C5—C1—C2—C3	0.8 (3)	C32—C33—C34—C39	−176.3 (3)
C13—C2—C3—C4	179.5 (3)	C39—C34—C35—C36	0.7 (5)
C1—C2—C3—C4	2.1 (3)	C33—C34—C35—C36	−176.8 (3)
C2—C3—C4—C5	−4.1 (3)	C34—C35—C36—C37	1.4 (5)
O1—C1—C5—C6	−0.8 (5)	C35—C36—C37—C38	−3.4 (5)
C2—C1—C5—C6	178.9 (3)	C35—C36—C37—C47	178.0 (3)
O1—C1—C5—C4	176.9 (3)	C36—C37—C38—C39	3.5 (5)
C2—C1—C5—C4	−3.4 (3)	C47—C37—C38—C39	−178.0 (3)
C3—C4—C5—C6	−178.1 (3)	C37—C38—C39—C34	−1.5 (5)
C3—C4—C5—C1	4.6 (3)	C35—C34—C39—C38	−0.6 (5)
C1—C5—C6—C7	176.5 (3)	C33—C34—C39—C38	177.0 (3)
C4—C5—C6—C7	−0.5 (6)	C28—C29—C40—C41	173.8 (3)
C5—C6—C7—C12	−175.7 (3)	C30—C29—C40—C41	−3.6 (6)
C5—C6—C7—C8	0.7 (5)	C29—C40—C41—C42	10.0 (5)
C12—C7—C8—C9	1.3 (5)	C29—C40—C41—C46	−167.0 (3)
C6—C7—C8—C9	−175.2 (3)	C46—C41—C42—C43	3.5 (5)
C7—C8—C9—C10	1.3 (5)	C40—C41—C42—C43	−173.6 (3)
C8—C9—C10—C11	−3.8 (5)	C41—C42—C43—C44	0.0 (5)
C8—C9—C10—C20	173.7 (3)	C42—C43—C44—C45	−2.4 (5)
C9—C10—C11—C12	4.1 (5)	C42—C43—C44—C51	172.6 (3)
C20—C10—C11—C12	−173.4 (3)	C43—C44—C45—C46	1.3 (5)
C10—C11—C12—C7	−1.7 (5)	C51—C44—C45—C46	−173.7 (3)
C8—C7—C12—C11	−1.1 (5)	C44—C45—C46—C41	2.2 (5)
C6—C7—C12—C11	175.6 (3)	C42—C41—C46—C45	−4.5 (5)
C1—C2—C13—C14	174.1 (3)	C40—C41—C46—C45	172.8 (3)
C3—C2—C13—C14	−3.0 (6)	C38—C37—C47—C49	23.2 (5)
C2—C13—C14—C15	10.0 (5)	C36—C37—C47—C49	−158.4 (4)
C2—C13—C14—C19	−169.6 (3)	C38—C37—C47—C50	−96.5 (4)
C19—C14—C15—C16	2.4 (5)	C36—C37—C47—C50	81.9 (4)
C13—C14—C15—C16	−177.2 (3)	C38—C37—C47—C48	144.1 (4)
C14—C15—C16—C17	0.1 (5)	C36—C37—C47—C48	−37.5 (5)
C15—C16—C17—C18	−2.2 (5)	C45—C44—C51—C53	−33.2 (4)
C15—C16—C17—C24	174.6 (3)	C43—C44—C51—C53	152.1 (3)
C16—C17—C18—C19	1.5 (5)	C45—C44—C51—C52	−155.1 (3)
C24—C17—C18—C19	−175.2 (3)	C43—C44—C51—C52	30.2 (4)
C17—C18—C19—C14	1.1 (5)	C45—C44—C51—C54	86.3 (4)
C15—C14—C19—C18	−3.0 (5)	C43—C44—C51—C54	−88.4 (3)
C13—C14—C19—C18	176.6 (3)	O3—C55—C56—C58	6.5 (3)
C11—C10—C20—C21	−147.0 (3)	C56^i^—C55—C56—C58	−173.5 (3)
C9—C10—C20—C21	35.7 (4)	O3—C55—C56—C57	−176.10 (15)
C11—C10—C20—C23	−24.8 (4)	C56^i^—C55—C56—C57	3.90 (15)
C9—C10—C20—C23	157.9 (3)	C58—C56—C57—C57^i^	167.2 (3)
C11—C10—C20—C22	94.1 (4)	C55—C56—C57—C57^i^	−9.8 (4)
C9—C10—C20—C22	−83.2 (4)	C55—C56—C58—C59	177.1 (3)
C18—C17—C24—C26	−36.4 (4)	C57—C56—C58—C59	0.4 (6)
C16—C17—C24—C26	147.1 (3)	C56—C58—C59—C64	24.2 (5)
C18—C17—C24—C27	83.0 (4)	C56—C58—C59—C60	−156.7 (3)
C16—C17—C24—C27	−93.6 (4)	C64—C59—C60—C61	−2.5 (5)
C18—C17—C24—C25	−158.0 (3)	C58—C59—C60—C61	178.3 (3)
C16—C17—C24—C25	25.4 (4)	C59—C60—C61—C62	1.8 (5)
O2—C28—C29—C40	1.9 (5)	C60—C61—C62—C63	0.3 (5)
C32—C28—C29—C40	−178.0 (3)	C60—C61—C62—C65	−177.1 (3)
O2—C28—C29—C30	179.8 (3)	C61—C62—C63—C64	−1.7 (4)
C32—C28—C29—C30	0.0 (3)	C65—C62—C63—C64	175.7 (3)
C40—C29—C30—C31	178.1 (3)	C60—C59—C64—C63	1.1 (5)
C28—C29—C30—C31	0.5 (3)	C58—C59—C64—C63	−179.7 (3)
C29—C30—C31—C32	−0.7 (3)	C62—C63—C64—C59	1.0 (5)
O2—C28—C32—C33	0.3 (5)	C61—C62—C65—C67	−31.6 (4)
C29—C28—C32—C33	−179.9 (3)	C63—C62—C65—C67	151.2 (3)
O2—C28—C32—C31	179.7 (3)	C61—C62—C65—C68	87.2 (4)
C29—C28—C32—C31	−0.4 (3)	C63—C62—C65—C68	−90.0 (3)
C30—C31—C32—C33	−179.9 (3)	C61—C62—C65—C66	−153.7 (3)
C30—C31—C32—C28	0.7 (3)	C63—C62—C65—C66	29.0 (4)

Symmetry codes: (i) −*x*+1, *y*, −*z*+1/2.
